# Supplementary material for: Structural and functional properties of the transporter SLC26A6 reveal mechanism of coupled anion exchange
Source: eLife. 2023 Jun 23;12:RP87178. doi: 10.7554/eLife.87178 (PMC10328499; doi:10.7554/eLife.87178)
Supplement: Transparent reporting form [file elife-87178-transrepform1.docx]

***eLife’s* transparent reporting form**

We encourage authors to provide detailed information *within their submission* to facilitate the interpretation and replication of experiments. Authors can upload supporting documentation to indicate the use of appropriate reporting guidelines for health-related research (see [EQUATOR Network](http://www.equator-network.org/%20)), life science research (see the [BioSharing Information Resource](https://biosharing.org/)), or the [ARRIVE guidelines](http://www.plosbiology.org/article/info:doi/10.1371/journal.pbio.1000412) for reporting work involving animal research. Where applicable, authors should refer to any relevant reporting standards documents in this form.

If you have any questions, please consult our Journal Policies and/or contact us: [editorial@elifesciences.org](mailto:editorial@elifesciences.org).

**Sample-size estimation**

- You should state whether an appropriate sample size was computed when the study was being designed
- You should state the statistical method of sample size computation and any required assumptions
- If no explicit power analysis was used, you should describe how you decided what sample (replicate) size (number) to use

Please outline where this information can be found within the submission (e.g., sections or figure legends), or explain why this information doesn’t apply to your submission:

- For all the described experiments, detailed information on number of replicates are provided in figure legends and in the source data files.
- For electrophysiology recordings, measurements were repeated with additional replicates not substantially deviating the average of the combined measurements. Replicates for recordings are documented within legends of Figures 1 A & B, Figure Supplement 1 G & H and Figure 5C.
- Replicates from proteo-liposome experiments refer to separate samples prepared from a single batch of reconstituted proteo-liposomes for Figures 1 D-H, Figure Supplement 1 C-E & F and Figures 5 E-G. Multiple reconstitutions were preformed from both separate purified protein and synthetic lipid preparations resulting in consistent data.
- The sample size for cryo-EM analysis was chosen to obtain the best possible resolution for each dataset. The detailed procedure is outlined in materials and methods section on cryo-EM data collection and image processing

**Replicates**

- You should report how often each experiment was performed
- You should include a definition of biological versus technical replication
- The data obtained should be provided and sufficient information should be provided to indicate the number of independent biological and/or technical replicates
- If you encountered any outliers, you should describe how these were handled
- Criteria for exclusion/inclusion of data should be clearly stated
- High-throughput sequence data should be uploaded before submission, with a private link for reviewers provided (these are available from both GEO and ArrayExpress)

Please outline where this information can be found within the submission (e.g., sections or figure legends), or explain why this information doesn’t apply to your submission:

- For proteo-liposome experiments, the number of replicates from individual experiments and reconstitutions can be found within the appropriate figure legend.
- For electrophysiology, replicates refer to individual transfected cells.
- No obvious outliers were encountered or omitted from the presented data. Solely outliers resulting from leaky membrane patches in electrophysiology recordings were excluded.
- The presented data is in accordance with eLife’s regulations and appropriate information regarding the number and type of replicates is provided in figure legends and in the source data files.

**Statistical reporting**

- Statistical analysis methods should be described and justified
- Raw data should be presented in figures whenever informative to do so (typically when N per group is less than 10)
- For each experiment, you should identify the statistical tests used, exact values of N, definitions of center, methods of multiple test correction, and dispersion and precision measures (e.g., mean, median, SD, SEM, confidence intervals; and, for the major substantive results, a measure of effect size (e.g., Pearson's r, Cohen's d)
- Report exact p-values wherever possible alongside the summary statistics and 95% confidence intervals. These should be reported for all key questions and not only when the p-value is less than 0.05.

Please outline where this information can be found within the submission (e.g., sections or figure legends), or explain why this information doesn’t apply to your submission:

- For proteo-liposome assays and thermostability data mean and SEM of technical replicates were calculated as described in the figure legends
- For cryo-EM maps, the resolution was estimated using the gold-standard Fourier shell correlation between two independently refined half-maps. For details, see material and method section

(For large datasets, or papers with a very large number of statistical tests, you may upload a single table file with tests, Ns, etc., with reference to sections in the manuscript.)

**Group allocation**

- Indicate how samples were allocated into experimental groups (in the case of clinical studies, please specify allocation to treatment method); if randomization was used, please also state if restricted randomization was applied
- Indicate if masking was used during group allocation, data collection and/or data analysis

Please outline where this information can be found within the submission (e.g., sections or figure legends), or explain why this information doesn’t apply to your submission:

No group allocation was performed.

**Additional data files (“source data”)**

- We encourage you to upload relevant additional data files, such as numerical data that are represented as a graph in a figure, or as a summary table
- Where provided, these should be in the most useful format, and they can be uploaded as “Source data” files linked to a main figure or table
- Include model definition files including the full list of parameters used
- Include code used for data analysis (e.g., R, MatLab)
- Avoid stating that data files are “available upon request”

Please indicate the figures or tables for which source data files have been provided:

Figure 1, Figure 1-figure supplement 1, Figure 5.
